# The effects of wakeful rest on memory consolidation in an online memory study

**DOI:** 10.3389/fpsyg.2022.932592

**Published:** 2022-10-25

**Authors:** Olivia King, Jessica Nicosia

**Affiliations:** ^1^Department of Psychological and Brain Sciences, Washington University in St. Louis, St. Louis, MO, United States; ^2^Charles F. and Joanne Knight Alzheimer’s Disease Research Center, Department of Neurology, Washington University School of Medicine, St. Louis, MO, United States

**Keywords:** wakeful rest, memory, online studies, consolidation, prolific

## Abstract

*Memory consolidation* is the process in which memory traces are strengthened over time for later retrieval. Although some theories hold that consolidation can only occur during sleep, accumulating evidence suggests that brief periods of wakeful rest may also facilitate consolidation. Interestingly, however, Varma and colleagues reported that a demanding 2-back task following encoding produced a similar performance to a wakeful reset condition. We tested whether participants’ recall would be best following a wakeful rest condition as compared to other distractor conditions, consistent with the extant wakeful rest literature, or whether we would replicate the finding by Varma and colleagues such that participants’ memory benefitted from both a rest and a 2-back task following encoding. Across two experiments, we used similar (Experiment 1) and the same (Experiment 2) encoding material as used the one by Varma and colleagues, employed a wakeful rest condition adapted for online testing, and compared participants’ recall across post-encoding conditions. In the first experiment, we used a between-subjects design and compared participants’ cued recall performance following a period of wakeful rest, a 2-back task, or a rest + sounds condition. The second experiment more closely replicated the experimental design used by Varma and colleagues using a within-subjects manipulation. Ultimately, our findings more consistently aligned with the canonical wakeful rest finding, such that recall was better following the rest condition than all other post-encoding conditions. These results support the notion that wakeful rest may allow for consolidation by protecting recently encoded information from interference, thereby improving memory performance.

## Introduction

Memory consolidation is broadly defined as the process in which memory traces become less susceptible to interference and are strengthened across time for later retrieval (Wixted and Cai, 2014). Although some theories hold that consolidation occurs primarily during sleep, accumulating evidence suggests that brief periods of wakeful rest following encoding may similarly facilitate the consolidation of new memories (Wamsley, 2019).

The typical wakeful rest paradigm consists of an incidental encoding phase, an immediate retrieval phase, a wakeful rest or distractor phase, and a final delayed retrieval phase. The critical manipulation occurs after the immediate retrieval phase: participants either undergo ∼10 min of wakeful rest, where they rest alone in a quiet room without access to distracting materials, or perform a simple distractor task, such as a passive listening task or visual search task (e.g., Dewar et al., 2012). Finally, participants complete a delayed retrieval test for the initially encoded stimuli. Performance following the wakeful rest period is typically better than performance following the distractor task (see Wamsley, 2019 for a review). It has been suggested that wakeful rest may reduce forgetting by allowing memory consolidation processes to occur (Wixted, 2004; Dewar et al., 2009, 2010, 2012; Tambini et al., 2010).

However, there has been some inconsistency in the literature across different post-encoding tasks and stimuli (Dewar et al., 2012; Varma et al., 2018). Studies using distractor tasks such as spot the difference, picture naming, and facial recognition tasks have shown wakeful rest effects (Dewar et al., 2009, 2012; Mercer, 2015), whereas others using a cognitively demanding n-back task did not show a wakeful rest effect (Varma et al., 2017 and 2018). Thus, it is clear that further investigation is necessary to better understand the mechanisms and circumstances under which a wakeful rest effect is obtained.

Several potential mechanisms have been proposed to account for the wakeful rest effect, including protection against retroactive interference (Dewar et al., 2009), rehearsal (Millar and Balota, 2018), and reduced/suppressed autobiographical thinking (Varma et al., 2018). For example, consider the initial study by Dewar et al. (2009), which investigated the effect of wakeful rest in a sample of amnesic patients to better understand how wakeful rest may protect memories from retroactive interference. Participants studied a list of 15 standardized, aurally presented words (Snodgrass and Vanderwart, 1980) before a 9-min post-encoding block, including interference or wakeful rest condition. The interference condition (i.e., a picture-naming task where the participant was instructed to name the picture rather than the word superimposed on the picture) was introduced either early on, in the middle, or at the end of the 9-min block. The wakeful rest condition included no interference, and participants simply rested alone in the quiet testing room, free of any potentially interfering material with the dimmed lights. Dewar et al. (2009) found that, overall, participants recalled more material from encoding in the wakeful rest condition than in the interference conditions. Moreover, within the interference conditions, participants’ performance was better when the interference was introduced at the end of the post-encoding block than when it was introduced early on or in the middle. These results suggest that not only did participants’ memory benefit from the post-encoding wakeful rest period but also that delaying interference allowed for more consolidation to occur. Thus, Dewar et al. (2009) concluded that the wakeful rest period allowed for consolidation by protecting recently encoded information from interference boosted the strength of the memory traces, and ultimately improved memory performance.

An alternative account of this pattern is that wakeful rest paradigms allow individuals to intentionally rehearse recently learned information (Millar and Balota, 2018; Wamsley, 2019). Specifically, it is possible that participants simply choose to explicitly rehearse encoded information in preparation for an upcoming retrieval task (even though participants often undergo incidental encoding tasks and are not informed about the final retrieval task in many wakeful rest studies). There are two major arguments against this rehearsal account. First, several studies have shown that wakeful rest elicits an equivalent benefit for “difficult-to-rehearse materials,” including non-words and non-English words (Dewar et al., 2014; Humiston and Wamsley, 2018). Second, although many wakeful rest studies include post-experiment questionnaires aimed at identifying participants who may have tried to prepare for the final memory test (Craig et al., 2014; Dewar et al., 2014), it appears that the exclusion of these individuals does not change the results. Although wakeful rest researchers have attempted to account for potential rehearsal confounds, further steps are necessary to determine which aspects of wakeful rest (explicit rehearsal or otherwise) may influence subsequent memory performance.

Varma et al. (2018) proposed an alternate explanation for the retroactive interference argument proposed by Dewar et al. (2009). Specifically, Varma et al. (2018) argued that studied materials are susceptible to being forgotten when consolidation is interrupted by “novel memory encoding or retrieval, associated with sensory stimulation from the environment or autobiographical thinking.” Drawing upon work by Wixted (2004) and Mednick et al. (2011), the authors suggest that retroactive interference occurs specifically when novel encoding usurps limited hippocampal resources that would otherwise be engaged in consolidation processes. Furthermore, they suggest a tradeoff between resources allocated toward memory consolidation and novel encoding processes (such as attending to the current environment/stimuli and maintaining a logical stream of thought). Under this assumption, Varma et al. (2018) argued that consolidation only suffers whenever the post-encoding period is filled with *novel episodic memory processing*, such as autobiographical thinking, and that limiting such thoughts during a period of wakeful rest could “free up” episodic memory resources for consolidation and thereby reduce interference effects.

Varma et al. (2018) investigated this possibility by comparing the efficacy of a standard wakeful rest period to a 2-back condition, where participants performed a 2-back working memory task, and to rest + sounds condition including sound cues (e.g., the sound of a dog barking causes one to think of their childhood pet) meant to trigger autobiographical thoughts (memories of personal events or experiences and future scenarios). Varma et al. (2018) hypothesized that the promotion of autobiographical thinking during the rest + sounds condition would be detrimental to memory consolidation and, therefore, final memory performance compared to both the rest (wakeful rest) condition and the 2-back condition while performing the 2-back task would suppress these autobiographical thoughts, benefiting memory performance. They compared participants’ recognition memory performance for information encoded before either the rest, 2-back, or rest + sounds post-encoding conditions. Remarkably, their results indicated that memory performance in the rest and 2-back conditions did not differ, while performance in the rest + sounds condition was significantly lower. Thus, memory consolidation appeared to occur in the demanding 2-back condition while being blunt in the other conditions. The authors suggested that only the promotion of autobiographical thoughts (i.e., in the rest + sounds condition) disrupted the consolidation process and that the 2-back task shielded participants from incurring autobiographical thoughts. However, the results of Varma et al. (2018) clearly contradicted other wakeful rest studies, which have shown that doing any demanding task (e.g., spot the difference task, picture naming, and facial recognition) post-encoding may disrupt consolidation as compared to wakeful rest. Thus, exploring the mechanisms involved in a successful wakeful rest condition is necessary.

## The current study

As the wakeful rest literature rapidly gains traction and the potential for application broadens, it is critical to understand the robustness of published results and potential boundary conditions of the wakeful rest phenomenon. Therefore, the present set of studies tested whether participants’ recall would be best following a wakeful rest condition as compared to the other distractor conditions, consistent with the extant wakeful rest literature or the findings presented by Varma et al. (2018) of a memory benefit in both the rest and 2-back conditions, compared to the rest + sounds condition. Across two experiments, we used similar (Experiment 1) and the same (Experiment 2) encoding material as that used by Varma et al. (2018), employed a wakeful rest condition adapted for online testing, and compared participants’ recall across post-encoding conditions. In the first experiment, we used a between-subjects design and compared participants’ cued recall performance following a period of wakeful rest to a 2-back task or a rest + sounds condition. The second experiment more closely replicated the experimental design used by Varma et al. (2018), such that we employed a within-subjects manipulation and used the exact stimuli (translated into English) as reported by Varma et al. (2018). Foreshadowing our findings, although we were ultimately unable to replicate the exact results by Varma et al. (2018), we did find a similar general pattern with the within-subjects design (Experiment 2), such that memory performance was worse when the rest followed encoding + sounds condition as compared to the rest and 2-back conditions. Interestingly, however, results from the between-subjects experiment (Experiment 1) were more consistent with the canonical wakeful rest finding, such that recall was better following the rest condition than all other conditions (i.e., 2-back, rest + sounds).

## Experiment 1

Our first experiment aimed to conceptually replicate the study by Varma et al. (2018) using a between-subjects design and a wakeful rest paradigm adapted for online testing. Specifically, participants first performed an incidental encoding task where they studied picture-word pairs, followed by a 9-min post-encoding period filled with either (1) a wakeful rest period, (2) a 2-back task, or (3) a rest + sounds condition, which played sound cues to trigger autobiographical memories. All participants, except for those in the 2-back condition, performed a brief 1-min “washout” period following the post-encoding task where they performed the Stroop task. The 2-back task was not followed by a Stroop washout period because the purpose of the washout period was to negate any potential refreshing effects of the rest and rest + sounds conditions. Given that the 2-back task was attentionally demanding (similar to the Stroop task), we chose not to include a Stroop washout period after. Finally, participants received a cued recall test at the end of the experiment. The cued recall test included pictures from encoding, which were the cues to recall the words with which the pictures were paired. A between-subjects design was used such that participants would only undergo one final retrieval period rather than four, as done by Varma et al. (2018), where participants underwent an immediate recall task after each encoding period and a final recall task. In this way, compared to the study by Varma et al. (2018), the participants in the present Experiment 1 should be less likely to rehearse the items in anticipation of an unexpected final test. We hypothesized that, consistent with the extant wakeful rest literature, participants’ recall would be best after the wakeful rest condition compared to the other conditions. The important issue is whether we would replicate the findings by Varma et al. (2018), a memory benefit in the 2-back task compared to the rest + sounds condition.

### Methods

#### Participants

Participants were recruited from the Younger Adult Subject Pool at Washington University in St. Louis and compensated at a rate of 1 credit/h. Inclusion criteria included native English-speaking ability, normal or corrected-to-normal vision, and access to a computer with the Google Chrome web browser. Participants were not allowed to participate in more than one of the present studies. Studies were posted to Washington University in St. Louis’ Sona System one at a time and run sequentially. Because different numbers of participants randomly signed up per condition, each condition had slightly different numbers of participants: rest (*N* = 52), 2-back (*N* = 52), and rest + sounds (*N* = 50). Assuming η_p_^2^ = 0.19 in the study by Varma et al. (2018) and adjusting for the between-subjects nature of our design, we aimed to recruit at least 50 participants for each condition for adequate power (1-β = 0.95).

#### Materials

Stimuli consisted of a set of 52 picture-word pairs with pictures drawn from the study by Oudiette et al. (2013) and words drawn from the study by Varma et al. (2019). The pictures were of everyday, concrete objects. The word stimuli consisted of adjectives that ranged from three to nine letters in length (*M* = 6.06 letters) and ranged in subtitle word frequency [SUBTL frequency norms, a word frequency measure based on American English television and film subtitles; Brysbaert and New, 2009, from 0.22 to 545.18 (*M* = 49.12)]. Autobiographical sounds used during the rest + sounds conditions were drawn from the study by Hall et al. (2014) and deliberately selected to avoid semantic similarity to the picture-word pairs.

All experimental programs were written in PsychoPy (Peirce et al., 2019), hosted on Pavlovia,^1^ and posted on the Washington University in St. Louis’ Sona System^2^ for recruitment.

#### Procedure

**FIGURE 1:**
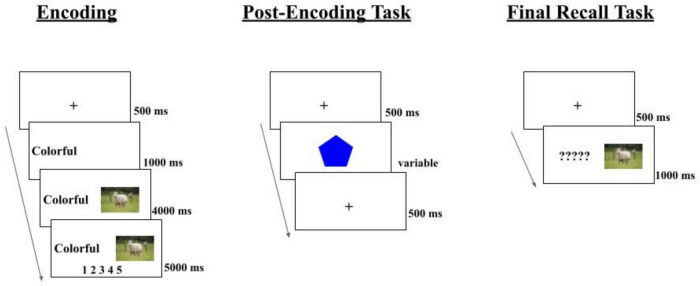
Experiment 1 procedure. The rest task is displayed as the post-encoding task in this figure, and its variable interval was 3–42 s.

A general overview of the experimental procedure is provided in Figure 1. In the rest + sounds condition, participants received an auditory check to confirm that the sound was working and that they could adequately hear and comprehend audited sounds/instructions. Participants visually presented the following directions during the auditory check: “This is an audio check. Please follow the instructions being presented auditorily. If you cannot hear the audio check, you will not be able to complete this experiment. Please close the tab.” Participants then received auditory instructions stating, “This is an audio check. Please take a moment to adjust the volume to a comfortable setting. Once the experiment begins, please refrain from adjusting the volume. This is critical for the experiment. If you can hear this message at a comfortable volume, please press the ‘Q’ key on your keyboard to move on.” These instructions were repeated (up to three times) until the participant responded with the correct key response (pressing Q). If the participant pressed Q, the experiment would commence. If the participant did not press Q before the auditory instructions had been repeated three times, the experiment would end (Nicosia, 2022).

As described earlier and shown in Figure 1, each participant performed three tasks during the experiment. First, participants performed an incidental encoding task. During each encoding trial, participants were asked to create an imaginative association between the word (e.g., “colorful”) and the picture (e.g., sheep). The word was presented on the screen for a fixed duration of 1,000 ms before the picture was presented, along with the word, for a fixed duration of 4,000 ms. Then, participants were asked to rate the vividness of their imagined association on a scale from 1 to 5 (i.e., not vivid-very vivid) by using the mouse to click along the scale within the next 5,000 ms. After 5,000 ms had elapsed, the next trial began with a fixation cross displayed for 500 ms. This was kept constant regardless of when participants responded.

Next, participants performed one of three post-encoding conditions, each lasting for ∼9 min: rest, 2-back, or rest + sounds. Participants performed a modified version of the Shapes task in the rest condition adapted from the study by O’Callaghan et al. (2015). Participants were given these instructions: “This portion of the study is looking at relaxation. Please look at the computer screen and try to relax with your eyes open while attending to the shapes.” This screen was displayed until participants pressed the spacebar to advance. Shapes were used in the rest condition to provide minimally demanding stimuli for participants to attend to. Because of the online nature of the present study, we wanted to ensure that participants were at least looking at the screen, rather than possibly interfering with material, during the wakeful rest period.

In the 2-back condition, participants were instructed: “In this portion of the experiment, you will see a digit (1–5) presented on the screen. If this number is the same as the one displayed two trials earlier, press the ‘j’ key; otherwise, press the ‘f’ key. Please do the task as quickly and accurately as possible. Press Space to continue.” For each trial, a random number (between 1 and 5) was displayed in the middle of the screen for 3,000 ms or until the participant responded. Also displayed on the screen was a reminder of the correct key-mapping: “Press ‘j’ if the digit is the same as two trials ago; otherwise, press ‘f.”’ After a response or 3,000 ms had elapsed, whichever came first, the next trial began with a fixation cross displayed for 500 ms. During this condition, participants engaged in 680 trials, half of which were the same as two trials earlier, while the other half of the trials were different. Participants were not given trial-by-trial feedback to minimize disruption in the continuous 2-back task.

The design of the rest + sounds condition was adapted by Varma et al. (2018). In this condition, participants received the same instructions as in the rest condition and performed the rest task. Additionally, however, 19 sounds (every four seconds in duration) were presented throughout the duration of the post-encoding condition. These stimuli consisted of sounds encountered in everyday life (e.g., “alarm clock,” “ducks,” “camera shutter,” etc.) that may trigger memories from one’s personal past or the imagination of a future scenario but did not share any semantic content with the encoding stimuli. Participants were instructed to rest quietly while sounds were played. No instructions were given to identify the sounds or engage in any autobiographical thinking during this period.

After the post-encoding task, participants in all conditions completed a questionnaire aimed at assessing their thoughts during the post-encoding task. First, participants were asked how demanding they found the post-encoding task they participated in. Participants responded on a scale from 1 to 5, with one representing “not at all demanding” and five representing “very demanding.” Next, participants were presented with the following questions, one at a time: “What percentage (%) of your thoughts during the [post-encoding task specified here] was related to the [post-encoding task specified here]?” “What percentage (%) of your thoughts during the [post-encoding task specified here] was related to past/future events?” “What percentage (%) of your thoughts during the [post-encoding task specified here] was related to the picture-word pairs you rated earlier?” “What percentage (%) of your thoughts during the [post-encoding task specified here] was related to something else not mentioned?” Participants responded on a scale from 0% of the time to 100% of the time.

As mentioned previously, all participants (except those in the 2-back condition) then completed a 1-min round of the Stroop task. This task minimizes any refreshing or carry-over effects that may differ between the conditions (e.g., Dewar et al., 2014; Millar and Balota, 2018). Any benefit of wakeful rest on memory consolidation should be present after this 1-min washout. Participants were presented with instructions that read: “In this portion of the experiment, you will see words (‘RED,” ‘GREEN,’ and ‘BLUE”) presented in different colored inks. The ink color may be the same or different from the color of the word. Please press the key corresponding to the INK COLOR and do your best to IGNORE the word. Press SPACE to continue.” This screen was presented until participants pressed the spacebar to advance. Participants performed 72 trials consisting of red, blue, and green word and color stimuli, 36 of which were congruent (i.e., color and word matching) and 36 were incongruent (i.e., color and word not matching). Each trial was presented for 4,000 ms until participants pressed a key response. Participants were not provided any feedback on their responses. After pressing a key response or 4,000 ms had passed, the next trial began with a 200 ms fixation cross.

After the post-encoding period, participants performed a cued recall task of the initially encoded picture-word pairs. Participants were given these instructions: “Now, you will be presented with a picture from earlier in the experiment. Please recall the WORD paired with the picture from earlier in the experiment. Please type your response using the keyboard and press ENTER after you are done to move on. Please press SPACE to begin.” This screen was presented until participants pressed the spacebar to advance. After typing their response and pressing the return key, or 10,000 ms had expired, whichever came first, the next trial began with a fixation cross displayed for 500 ms. Once the cued recall task was finished, participants were then thanked for their participation and prompted to press the spacebar to complete the experiment.

### Results

For all results reported, statistical significance was set at *p* < 0.05, a two-tailed test, unless otherwise noted. Effect sizes of eta squared (η^2^, Olejnik and Algina, 2003) are reported for significant *F* tests and Cohen’s *d* (*d*, Cohen, 1988) for significant *t*-tests. Adjusted degrees of freedom are reported such that unequal variances were assumed, and the Welsh approximation was applied.

First, analyses on recall accuracy are presented to test the main hypothesis that participants’ recall would be best following the wakeful rest condition compared to the other conditions. Second, participants’ self-reported difficulty ratings are examined to test whether the demanding nature of the task performed during the retention interval could explain the difference in wakeful rest effects found across conditions. As mentioned, we hypothesized that, consistent with the extant wakeful rest literature, participants’ recall would be best following the wakeful rest condition compared to the other conditions. The important issue is whether we would replicate the finding by Varma et al. (2018) of a memory benefit in the 2-back task compared to the rest + sounds condition.

#### Recall accuracy

**FIGURE 2:**
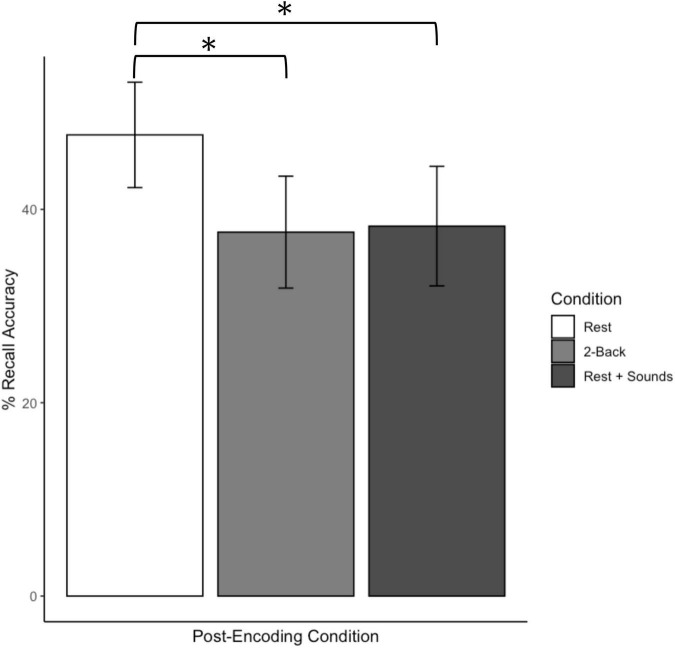
Recall accuracy as a function of post-encoding conditions for Experiment 1. Error bars represent confidence intervals. *Indicates *p* < 0.05.

Percent correct recall accuracy as a function of the post-encoding task for Experiment 1 is displayed in Figure 2. Overall, participants in the rest (wakeful rest) condition correctly recalled more words than participants in the other conditions. These patterns were confirmed by a one-way analysis of variance (ANOVA), which yielded an effect of post-encoding condition, *F*(2, 151) = 3.83, *p* = 0.02, η^2^ = 0.05. Planned *t*-tests confirmed that recall was significantly better in the rest condition as compared to the 2-back condition, *t*(101.64) = 2.54, *p* = 0.01, *d* = 0.50, and the rest + sounds condition, *t*(97.95) = 2.30, *p* = 0.02, *d* = 0.46. Recall that the 2-back and rest + sounds conditions did not differ significantly *p*s = 0.88.

#### Difficulty ratings

**FIGURE 3:**
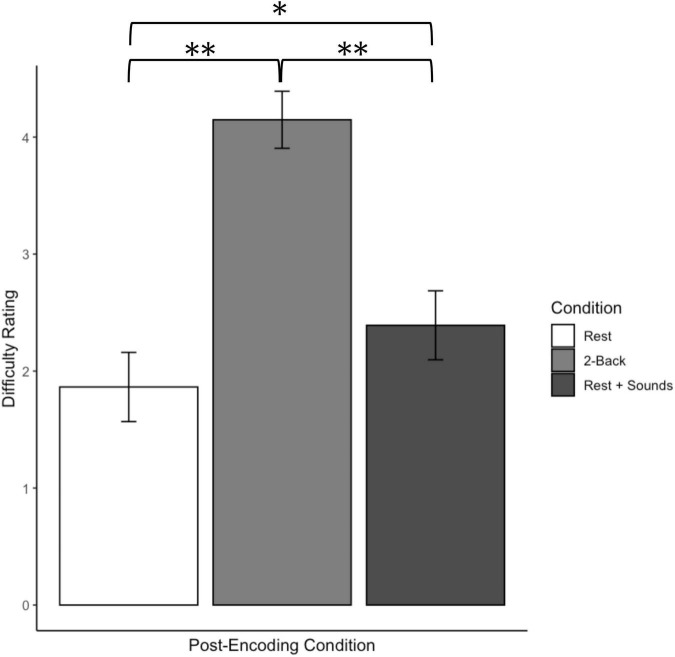
Participants’ self-reported difficulty ratings of the post-encoding condition tasks function as a post-encoding task for Experiment 1. Error bars represent confidence intervals. *Indicates *p* < 0.05 and ^**^Indicates *p* < 0.01.

Participants’ self-reported difficulty ratings of the post-encoding condition tasks as a function of a post-encoding task for Experiment 1 are displayed in Figure 3. Overall, participants rated the 2-back condition as the most difficult, followed by the rest + sounds condition, and the rest condition as the least demanding. These patterns were confirmed by a one-way analysis of variance (ANOVA) which yielded an effect of post-encoding condition, *F*(2, 151) = 75.02 *p* < 0.001, η^2^ = 0.50. Follow-up *t*-tests confirmed that participants found the rest condition less difficult than the both 2-back condition, *t*(98.44) = 11.97, *p* < 0.001, *d* = 2.35, and the rest + sounds condition, *t*(99.97) = 2.54, *p* = 0.01, *d* = 0.50. Additionally, participants reported the 2-back condition to be more difficult than the rest + sounds, *t*(95.90) = 9.22, *p* < 0.001, *d* = 1.83.

### Discussion

To summarize the results of Experiment 1, using a between-subjects version of the Varma et al. (2018) paradigm adapted for online testing, we found the canonical wakeful rest effect such that participants in the rest condition recalled more information than participants in either the 2-back or rest + sounds conditions. Additionally, consistent with the hypothesis put forth in the extant wakeful rest literature, participants rated all of the “distractor” conditions as more demanding than the rest condition, suggesting that when any additional cognitive demand or potentially interfering information follows encoding, there is minimal-to-no benefit as in the wakeful rest condition.

It is important to note, however, that in contrast to simply presenting a fixation cross on the screen and asking participants (who completed the experiment on their personal computers outside of the controlled laboratory setting) to focus on the fixation for ∼9 min, the Shapes condition provided a minimally demanding stimulus to maintain participants’ attention. Indeed, in earlier pilot studies, we found this manipulation to be critical for maintaining participants’ engagement in the experiment. Pilot analyses corroborated the notion that the rest + sounds condition used here did not significantly differ in either recall accuracy, *t*(86.26) = 1.73, *p* = 0.088, or difficulty ratings, *t*(81.42) = 1.22, *p* = 0.23, from a fixation + sounds condition (where participants simply stared at a blank fixation cross), indicating that using the Shapes task as an online version of the wakeful rest condition was similarly minimally demanding as if participants were simply staring at a blank fixation cross. Interestingly, the difficulty rating was numerically higher for the fixation + sounds condition than the rest + sounds condition, whereas recall was slightly worse. Thus, it seems possible to achieve wakeful rest effects in online studies. However, simple modifications should be made to maintain participants’ engagement in the task while balancing potential differences between active and passive rest.

In contrast to the findings presented by Varma et al. (2018), participants’ recall was worse following the 2-back condition than following the rest condition. However, there were several critical differences between the design used by Varma et al. (2018) and the design used in Experiment 1. First, we used a between-subjects design to reduce the number of retrieval periods that participants would complete so that participants would be less likely to expect the final recall task. Second, participants incidentally encoded picture-word pairs and performed a cued recall task instead of intentionally encoding a word list and performing a free recall task. Third, with the exception of the 2-back task, participants received a Stroop task for a washout period. Finally, to make the task more suitable for the online testing format, we used the Shapes task instead of having participants stare at a blank screen (as discussed in the previous paragraph). Thus, because of these potentially critical differences in our experimental design, in Experiment 2, we sought to test a more directly comparable experimental design to the one used by Varma et al. (2018).

## Experiment 2

### Methods

#### Participants

Forty-two participants were recruited from the Younger Adult Subject Pool at Washington University in St. Louis and compensated at a rate of 1 credit/h. Inclusion criteria were the same as those in Experiment 1. Because of the within-groups nature of the present study, six different counterbalancing orders were used for the post-encoding tasks. Assuming η_p_^2^ = 0.19 in the study by Varma et al. (2018) and adjusting for the within-subjects nature of our design, we aimed to recruit at least 40 participants for adequate power (1-β = 0.95).

#### Materials

Stimuli consisted of 45 words used in the study by Varma et al. (2018). The word *stimuli* was translated from Dutch into English and consisted of commonly used nouns. As described by Varma et al. (2018), these words were chosen to have minimal semantic relatedness but similar word frequency and concreteness (see Varma et al., 2018 for additional information on the word stimuli). Autobiographical sounds used during the rest + sounds condition were the same as used in Experiment 1, drawn from the study by Hall et al. (2014), and selected to avoid semantic similarity to the words used. From the 45 words, three lists of 15 words were created for use in each section of the experiment.

All experimental programs were written in PsychoPy (Peirce et al., 2019), hosted on Pavlovia (see text footnote 1), and posted on the Washington University in St. Louis’ Sona System (see text footnote 2) for recruitment.

#### Procedure

**FIGURE 4:**
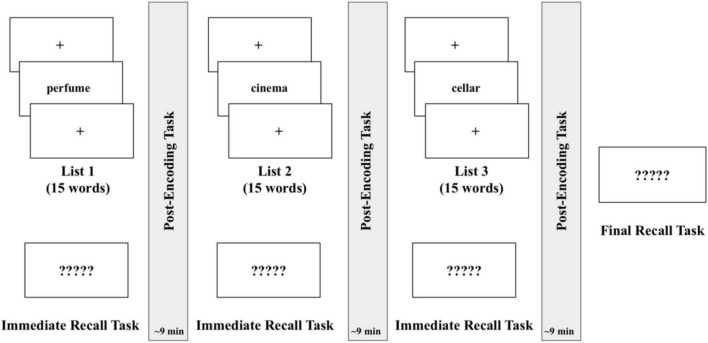
Experiment 2 procedure.

An overview of the Experiment 2 procedure is displayed in Figure 4. As in Experiment 1, all participants received an auditory check to confirm that the sound was audible at an appropriate level.

Following the study by Varma et al. (2018), the experiment was divided into three blocks, one for each condition (see Figure 4). Each block consisted of an encoding session, followed by an immediate recall test and a 9-min post-encoding period. Across the three blocks, the 9-min post-encoding period was filled with either a wakeful rest (rest task, as described in Experiment 1), a rest period interspersed with familiar sounds (rest + sounds condition, as described in Experiment 1), or a 2-back task (2-back condition, as described in Experiment 1), in a counterbalanced order across participants. There were no breaks between successive blocks. At the end of the third block, a surprise (delayed) free-recall task was administered. During both the immediate and delayed recall tests, participants were asked to recall as many words as possible in any order. Participants typed in their responses and clicked a button on the screen to advance to the next portion of the experiment when they felt they had recalled as many words as possible.

During the encoding sessions, a list of 15 words was presented visually, one word every 5,000 ms. Participants were instructed to memorize the upcoming word list and to expect a memory test immediately following the presentation. Instructions read as follows: “You will be shown a short list of words presented once. Please do your best to memorize the list of words. You will be tested on your ability to recall these words immediately following their presentation. Please press SPACE to continue.” After all 15 words had been presented, the immediate recall test was conducted to obtain an initial memory retention score. Next, the 9-min post-encoding interval began. One of the three post-encoding conditions was filled with the rest task, one with the 2-back task, and one with the rest + sounds condition (all described earlier). After undergoing three encoding, immediate recall, and post-encoding condition blocks, participants completed a surprise delayed recall task. Instructions read as follows: “Lastly, you will recall as many words from the ENTIRE EXPERIMENT as possible. Please type your response using the keyboard and press ENTER after you are done to move on. Click the blue button at the bottom of the screen when you feel you have recalled as many words as possible to end this portion of the experiment. Please press SPACE to begin.”

### Results

#### Immediate recall accuracy

Following the findings by Varma et al. (2018), we first examined whether immediate recall scores (the number of items correctly recalled during the immediate recall tasks) differed as a post-encoding condition. Consistent with the findings presented by Varma et al. (2018), the immediate recall was no different across post-encoding conditions. This pattern was confirmed by a one-way analysis of variance (ANOVA), which indicated that immediate recall scores (rest: *M* = 9.86 items correctly recalled, SD = 3.24; 2-back: *M* = 8.86, SD = 3.75; rest + sounds: *M* = 9.42, SD = 3.72) did not differ significantly between the three encoding blocks, *F*(2, 111) = 0.79, *p* = 0.45, η^2^ = 0.01, although recall for the 2-back condition items was numerically lower.

#### Delayed recall accuracy

We next examined whether delayed recall scores (the number of items correctly recalled during the delayed recall task) differed as a function of the post-encoding condition. There were no differences in delayed recall across post-encoding conditions. This pattern was confirmed by a one-way ANOVA, which indicated that delayed recall scores (rest: *M* = 6.65 items correctly recalled, SD = 3.55; 2-back: *M* = 5.42, SD = 4.01; rest + sounds: *M* = 5.33, SD = 4.22) did not differ significantly between the three encoding blocks, *F*(2, 111) = 1.51, *p* = 0.22, η^2^ = 0.02, although the performance on the rest condition items was numerically higher.

#### Proportional retention score

Following the findings presented by Varma et al. (2018), we calculated the “proportional retention score” for each condition. The number of words recalled during the delayed recall test was divided by the number originally recalled during the immediate recall test, yielding the proportional retention score. Additionally, consistent with the study by Varma et al. (2018), in cases where the delayed recall score exceeded the immediate recall score (which was true for 4 participants, 3 in the rest condition and 1 in the 2-back condition), the proportional retention score was capped at 1 (as done in the study by Varma et al., 2018). Inclusion of proportional retention scores greater than one (which occurred for four participants with non-capped scores ranging from 1.07 to 1.25) did not change (in statistical significance or magnitude) any of the results reported.

**FIGURE 5:**
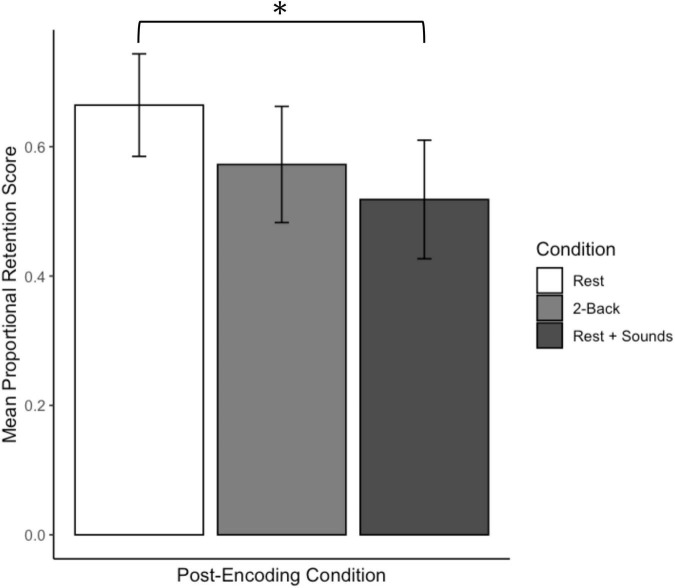
Participants’ proportional retention scores as a function of a post-encoding task for Experiment 2. Error bars represent confidence intervals. Asterisk (*) indicates *p* < 0.05.

Proportional retention scores as a function of a post-encoding task for Experiment 2 are displayed in Figure 5. Overall, participants in the rest (wakeful rest) condition correctly recalled more words than participants in the other conditions. However, consistent with the study by Varma et al. (2018), participants showed a benefit in both the rest and 2-back conditions compared to the rest + sounds condition. These patterns were confirmed by a two-way condition (rest, 2-back, rest + sounds) by order (counterbalance 1–6) mixed-factor ANOVA, which yielded a main effect of condition, *F*(2, 108) = 3.03, *p* = 0.04, η^2^ = 0.05. Follow-up *t*-tests confirmed that the proportional retention score was significantly better in the rest condition as compared to the rest + sounds condition, *t*(80.32) = 2.54, *p* = 0.01, *d* = 0.50, but that scores did not differ across the rest and 2-back conditions, *t*(80.75) = 1.54, *p* = 0.13, nor the 2-back and rest + sounds conditions, *t*(81.97) = 0.85, *p* = 0.40. There was also a condition by order interaction, *F*(10, 108) = 2.50, *p* = 0.01, η^2^ = 0.17, which indicated that the proportional retention score was better for the rest condition in the counterbalance orders when the rest condition occurred last. Consistent with that reported by Varma et al. (2018), this seems to indicate that there may be a slight advantage to memory retention in conditions that occur either at the beginning or the end, compared to the delay condition that occurs in the middle. However, the interaction results should be interpreted cautiously, so there were only seven participants within each counterbalance order.

### Discussion

The results indicated that participants’ proportional retention scores were better for words encoded before the rest and 2-back conditions than in the rest + sounds condition. However, also consistent with that reported by Varma et al. (2018), there were counterbalanced order effects, which showed that the effect was most robust when the rest condition was presented last. This somewhat complicates reconciling the results of Experiments 1 and 2, each having unique experimental design advantages. While Experiment 2 controls for individual differences in participants’ baseline memory performance because of the within-subjects design and proportional retention score, Experiment 1 better controls for potential rehearsal effects. Of course, one might argue that one possible solution for future studies would be to have participants participate in three sessions on separate days rather than complete all three conditions in a single session.

It is important to note that although there was a significant difference between the mean proportional retention score of the rest and the rest + sounds stimuli, the proportional retention score for the 2-back items was not significantly different from either the rest or the rest + sounds words. In comparison to the study by Varma et al. (2018), who found a significant difference between the 2-back and rest + sounds conditions, we did not replicate this finding. In this sense, we were able to replicate the general pattern of results by Varma et al. (2018), although the pairwise comparisons did not reach significance. In accordance with our initial hypothesis, we found a significant wakeful rest effect such that participants showed a higher proportional retention score for the rest condition than for the rest + Sound condition. However, the difference between the rest and 2-back conditions was insignificant.

There were a few critical differences in our conceptual replication of the findings by Varma et al. (2018), the first of which being that we used the Shapes task during the rest and rest + sounds conditions rather than a fixation cross to make the experiment more suitable for the online layout (see Experiment 1 discussion for further explanation). Additionally, during the 2-back task, participants were not given trial-by-trial feedback, and there was no performance threshold set, as done by Varma et al. (2018). Thus, the lack of feedback on the 2-back task may have contributed to participants’ slightly lower memory performance in that condition. Nevertheless, we aimed to more closely replicate the experimental design used by Varma et al. (2018) in Experiment 2 and indeed yielded results more closely in line with their hypothesis regarding the suppression of autobiographical thoughts to promote consolidation.

## General discussion

The results from the present study support findings consistent with the wakeful rest literature in that participants’ recall were best following a wakeful rest condition as compared to other distractor conditions. We compared participants’ recall following a wakeful rest condition to their recall following distractor conditions. The goal of this study was to test whether, consistent with the extant wakeful rest literature, participants’ recall would be better following wakeful rest only or whether we would replicate the findings by Varma et al. (2018) of a memory benefit in both the rest and 2-back conditions compared to the rest + sounds condition. Across two experiments, we employed a wakeful rest condition adapted for online testing and compared participants’ recall across post-encoding conditions. In the first experiment, we used a between-subjects design and compared participants’ cued recall performance following a period of wakeful rest to a 2-back task or a rest + sounds condition. The second experiment more closely replicated the experimental design used by Varma et al. (2018), such that we employed a within-subjects manipulation and used the exact stimuli (translated into English) as reported by Varma et al. (2018).

The results from Experiment 1 supported the canonical wakeful rest finding such that participants in the rest condition recalled more information from encoding than participants in either the 2-back or rest + sounds conditions. Indeed, consistent with the hypothesis commonly described in the wakeful rest literature, participants rated both “distractor” conditions as more demanding than the rest condition. Taken together with the recall accuracy data, the results of Experiment 1 suggest that any additional cognitive demand or potentially interfering information that follows encoding may prevent the beneficial effects of wakeful rest.

There were, however, several critical differences between the findings presented by Varma et al. (2018) and those in Experiment 1. First, Experiment 1 was conducted entirely online. Second, in an attempt to reduce participants’ anticipation of a final recall test, we used a between-subjects design that (1) reduced the number of retrieval periods that participants would complete and (2) had participants incidentally (rather than intentionally) encode picture-word pairs. Third, participants performed a cued recall task instead of a free recall task. Finally, to make the task more suitable for the online testing format, we used the Shapes task instead of having participants stare at a blank screen. Thus, in Experiment 2, we sought to test a within-subjects design comparable to that used by Varma et al. (2018).

The results of Experiment 2, although *closer* to the findings by Varma et al. (2018), still did not directly replicate the results presented by Varma et al. (2018). Specifically, the results indicated that participants’ proportional retention scores were better for words encoded before the rest and 2-back conditions than in the rest + sounds condition. Although there was a significant difference between the mean proportional retention score of the rest and the rest + sounds stimuli, the proportional retention score for the 2-back items was not significantly different from either the rest or the rest + sounds words.

In comparing the results of Experiments 1 and 2, it should be noted that participants incidentally encoded stimuli in Experiment 1, whereas stimuli were intentionally encoded in Experiment 2. While Experiment 2 controls for individual differences in participants’ baseline memory performance because of the within-subjects design and proportional retention score, Experiment 1 better controls for potential rehearsal effects *via* incidental encoding.^3^ Additionally, differences in encoding and retrieval difficulty across Experiments 1 and 2 may have impacted the results. Specifically, 52 picture-word pairs were incidentally encoded and then retrieved *via* cued recall. In Experiment 2, participants intentionally encoded 15 words and retrieved this information *via* free recall. It is possible that encoding and/or retrieval difficulty may modulate wakeful rest effects and should be investigated in future studies (indeed, see Schapiro et al., 2018; Poskanzer et al., 2021 for evidence suggesting more weakly encoded information may be more highly prioritized for hippocampal replay during post-learning rest).

Taken together, both Experiments 1 and 2 provide more support in favor of the canonical wakeful rest effect, although each has its unique advantages and limitations. There were, however, several critical differences between the results in Experiment 2 and those presented by Varma et al. (2018). First, like Experiment 1, Experiment 2 was conducted entirely online. Second, as discussed, we used the Shapes task during the rest and rest + sounds conditions rather than a fixation cross to make the experiment more suitable for the online testing environment. Third, during the 2-back task, participants were not given trial-by-trial feedback, and there was no performance threshold set, as done by Varma et al. (2018). Nevertheless, we aimed to more closely replicate the experimental design used by Varma et al. (2018) in Experiment 2. We indeed yielded results more closely in line with their hypothesis (at least compared to Experiment 1) regarding suppressing autobiographical thoughts to promote consolidation. Of course, additional work is necessary to fully understand the boundary conditions under which one can produce a wakeful rest effect.

The present study presents several key conclusions: First, we replicated the canonical wakeful rest effect in an online testing format. This may be useful for future studies or applications seeking to boost memory consolidation for individuals from the comfort of their own homes. Second, these results suggest that, contrary to some theories of memory consolidation, memory consolidation can occur during brief periods of wakeful rest when other demanding processes are not presently engaged. Although additional investigations are certainly warranted, the present study’s results support the hypothesis that interference reduction rather than the suppression of autobiographical memories may be one of the critical mechanisms through which wakeful rest effects are observed.

## Author’s note

Aspects of this article were presented at Washington University in St. Louis as a final project by OK.

## Data availability statement

The raw data supporting the conclusions of this article will be made available by the authors, without undue reservation.

## Ethics statement

The studies involving human participants were reviewed and approved by the Washington University in St. Louis Institutional Review Board. Written informed consent for participation was not required for this study in accordance with the national legislation and the institutional requirements.

## Author contributions

OK and JN conceptualized the study, programmed the experiments, analyzed the data, and wrote up the results. Both authors contributed to the article and approved the submitted version.

## Footnotes

1
pavlovia.org

2
wupsych.sona-systems.com

3 It may be important to note that in memory consolidation studies examining the effects of sleep on memory performance, sleep effects are often only evident with intentional encoding when the material is deemed important for future use. Specifically, evidence of preferential consolidation comes from studies indicating that emotional salience (Hu et al., 2006; Sterpenich et al., 2009), intention to remember (Wilhelm et al., 2011; van Dongen et al., 2012), and anticipation of a future reward for correct remembering (Fischer and Born, 2009) all enhance consolidation during sleep.
